# Supplementary material for: Concurrent Targeting of Expressive Vocabulary and Speech Comprehensibility in Pre-Schoolers with Developmental Language Disorder and Phonological Speech Sound Disorder Features: A Survey of UK Practice
Source: Children (Basel). 2025 Nov 18;12(11):1568. doi: 10.3390/children12111568 (PMC12650986; doi:10.3390/children12111568)
Supplement: Supplementary file 1 [file children-12-01568-s001.zip › Supplementary material S1.pdf]

Supplementary materials S1: GRIPP 2 short form

**Section and item**	**Where stated within the article**	**Further elaboration/additional information**
**Aim:** Aim of PPI in the study	2.1 section of the methodology	Enhance ‘implementation’ value of the work, reflect real world issues.
**Methods:** Clear description of the methods used for PPI in the study	2.1 section of the methodology	Project steering group- individual and whole group meetings, recorded via an impact log.
**Study results:** Outcomes- reported results of PPI in the study, including positive and negative outcomes	2.1 section of the methodology	Project steering group- Alterations to the survey prior to piloting. Integrated key discussion points (e.g. readiness for therapy) into the discussion section.
**Discussion and conclusions:** Outcomes—Comment on the extent to which PPI influenced the study overall.	2.1 section of the methodology	Positive: Diverse views reflected within the discussion, survey content which could be easily interpreted by respondents.

Describe positive and negative effects		Negative: Researchers have not had the opportunity to feedback the current study findings to the parents and clinicians who were involved in the pre-study PPI/engagement work yet.
**Reflections/critical perspective:** Comment critically on the study, reflecting on the things that went well and those that did not, so others can learn from this experience	Discussion-4.5 limitations	N/A
