# Supplementary material for: Concurrent Targeting of Expressive Vocabulary and Speech Comprehensibility in Pre-Schoolers with Developmental Language Disorder and Phonological Speech Sound Disorder Features: A Survey of UK Practice
Source: Children (Basel). 2025 Nov 18;12(11):1568. doi: 10.3390/children12111568 (PMC12650986; doi:10.3390/children12111568)
Supplement: Supplementary file 1 [file children-12-01568-s001.zip › Supplementary material S2.pdf]

Supplementary material S2: Steering group notes

Date	Event	Discussion topic: Survey questions	Actions + timeframe	Longer term impact and date reflected on
**22/5/23**	**1:1 discussion with SG member 1**	Rationale for phonological awareness (PA) activity choice- is it necessary? Long winded if not essential. Case description- some clinicians might worry about not being able to comment on different sub-groups and children with different characteristics (e.g. bilingual).	Take out open question for PA activities. Re-word case example.	Whole group meeting (June 23)<br>+<br>1:1 meetings following data collection
**23/5/23**	**1:1 discussion**	Ranking of PA activities- seems strange, might not be familiar to SLTs. Might get more from asking an open question so clinicians can provide their own	Replace PA ranking with 'what aspects of PA would you target?'	

	**with SG member 2**	answer. Case description-what about tailoring to differing profiles within this core description? Clinicians will not be aware of us looking at this in the future, so it needs to be clearly stated.	Re-word instructions, emphasise will look at adaptations for more individualistic profiles in the future.	
