# Supplementary material for: Concurrent Targeting of Expressive Vocabulary and Speech Comprehensibility in Pre-Schoolers with Developmental Language Disorder and Phonological Speech Sound Disorder Features: A Survey of UK Practice
Source: Children (Basel). 2025 Nov 18;12(11):1568. doi: 10.3390/children12111568 (PMC12650986; doi:10.3390/children12111568)
Supplement: Supplementary file 1 [file children-12-01568-s001.zip › Supplementary material S3.pdf]

Supplementary material S3: Survey questions

Introduction page

Identifying the content, format and process of delivery of a combined intervention for expressive vocabulary and speech comprehensibility (intelligibility): an anonymous online survey of UK clinical practice

City, University of London Ethics reference number: **ETH2223-2120**

Please read our [Participant information sheet]

This survey is part of a larger scale intervention development project, aiming to develop an intervention for pre-school children with co-occurring Speech Sound Disorder (SSD)/Developmental Language disorder (DLD) features (note: they may not have a formal diagnosis yet).

This survey aims to identify how you would provide an intervention for a child with this profile, where you are aiming to increase both their expressive vocabulary and speech intelligibility (these outcomes were selected by clinicians and parents of children with this profile within previous stakeholder engagement work).

We will use the information you provide to guide what this new intervention could look like.

Your responses are anonymous. There are no right or wrong answers. The most important thing is that you answer honestly.

The survey should take you approximately 20-30 minutes to complete. Your participation in this research is voluntary. You have the right to withdraw at any point during the study. However, data collected before the point of withdrawal would still be analysed. If you have any questions, please email them to the Principal Investigator of this study -------.

Thank you in advance for being a part of developing this new intervention.

Survey opening page: Core child characteristics

Please do not worry about making written notes. ***You will be given reminders*** *of the child's core characteristics during the survey.*

Age: between 3:0 and 4:11 years.

Clinical profile: features of a consistent phonological SSD and DLD.

Impact: the child is struggling to use a range of words in everyday life. The words they do have are often unintelligible to the people around them.

They may or may not have receptive language needs, but speech intelligibility and expressive vocabulary are currently their main areas of need.

Your intervention: you are providing a COMBINED intervention to target both expressive vocabulary AND speech intelligibility.

Please note: speech intelligibility might be improved through environmental strategies, as well as by developing the accuracy of their speech production.

Please base your responses on what **you** would do, rather than on what your service might currently offer.

The survey will start on the next page

<u>Survey page 1</u>

<u>Reminder</u>
Age: **3:0-4:11 years**
Profile: **(consistent) phonological SSD** and features of **DLD**.
Intervention: COMBINED to target both **Expressive vocabulary + speech intelligibility.**

Speech intelligibility might be improved through environmental strategies or by developing the accuracy of speech production.

You may think “my answers depend on…”. That’s ok. Just give the answer you think fits best.

Please base your responses on what you would do, rather than on what your service might currently offer.

In this first part of the survey, we will be asking you about intervention targets.

---

1. What would you target to improve **speech intelligibility** within this combined intervention? *when considering that the child's difficulties might make them suitable for any of the approaches below)*

1-highest priority; 5-lowest priority
(drag and drop your responses into place)
______ No specific sounds- increase in general awareness/use of sounds
______ Specific sounds according to the child’s profile- guided by what is having the most impact on them being understood by others
______ Specific sounds according to the child’s profile- guided by typically developing norms (i.e. the sounds the child "should" already have)
______ Specific sounds according to the child’s profile- guided by what the child is most stimulable for

2. What would you target to improve **expressive vocabulary** within this combined intervention? (*when considering that the child's difficulties might make them suitable for any of the approaches below)*

1-highest priority; 4-lowest priority

______ No specific vocabulary/word groups- focus on overall vocabulary enrichment
______ Specific vocabulary/word groups- guided by typically developing norms
______ Specific vocabulary/word groups- guided by vocabulary of importance to the child's everyday life
______ Specific vocabulary/word groups-guided by the particular sound properties within the words

3. How would you initially format your speech and vocabulary targets within this combined intervention?

1-highest priority; 5-lowest priority

______ I would integrate both speech AND vocabulary targets into the same activities within a therapy session
______ I would alternate between one activity targeting speech followed by one activity targeting vocabulary within a therapy session
______ For the first half of the intervention I would target vocabulary, then the latter half focus I would target speech. (e.g. in a 6 week block- the first 3 sessions are for vocabulary, the following 3 sessions are for speech).
______ For the first half of the intervention I would target speech, then the latter half focus I would target vocabulary. (e.g. in a 6 week block- the first 3 sessions are speech, the following 3 sessions are for vocabulary).
______ I would alternate between a session for speech targets and a session for vocabulary targets for the duration of the intervention

4. Would you also target phonological awareness within this integrated intervention for expressive vocabulary and speech intelligibility?

Yes- I would target general phonological awareness (not sound/process specific)

Yes- I would incorporate the specific sounds/processes that the child struggles with into these phonological awareness activities

No

Skip To: End of Block If Would you also target phonological awareness within this integrated intervention for expressive v... = No

15. Please label aspects of phonological awareness you might work on

____

Survey page 2
Reminder
Age: **3:0-4:11 years**
Profile: **(consistent) phonological SSD** and features of **DLD**.
Intervention: COMBINED to target both **Expressive vocabulary + speech intelligibility.**

Speech intelligibility might be improved through environmental strategies or by developing the accuracy of speech production.

You may think “my answers depend on…”. That’s ok. Just give the answer you think fits best.

lease base your responses on what you would do, rather than on what your service might currently offer.
You are a quarter of the way through the survey. In the next part, we will be asking you about intervention techniques for expressive vocabulary.

6. What intervention techniques would you consider for improving **expressive vocabulary** within this integrated intervention with speech intelligibility?

	Yes	No
Expansion: Repeating what the child says back to them and adding a word to it	○	○
Cloze procedure: (e.g. the adult omits a word from a common rhyme/story- the child then has the opportunity to complete it)	○	○
Labelling: labelling/modelling vocabulary in context (e.g. with a corresponding picture, object or action)	○	○
Time delays: (e.g. modelling and then giving an 'expectant pause' for the child to imitate)	○	○

Focused auditory stimulation: frequent repetition of specific vocabulary	○	○
Broad target recasts (using a language recast if the child's speech is relatively clear, and a speech recast if unclear speech is the main issue)	○	○
Choices: also known as 'forced alternatives' (e.g. "do you want the APPLE or the BANANA?")	○	○

7. Would you prioritise any of **your selected** techniques over the others?

- ◯ Yes
- ◯ No

Display This Question:

If Would you prioritise any of your selected techniques over the others? = Yes

Carry Forward Selected Choices from "What intervention techniques would you consider for improving expressive vocabulary within this integrated intervention with speech intelligibility?"

8. If yes to the above, how would you prioritise your selected techniques?

1-highest priority
_______ Expansion: Repeating what the child says back to them and adding a word to it
_______ Cloze procedure: (e.g. the adult omits a word from a common rhyme/story- the child then has the opportunity to complete it)
_______ Labelling: labelling/modelling vocabulary in context (e.g. with a corresponding picture, object or action)
_______ Time delays: (e.g. modelling and then giving an 'expectant pause' for the child to imitate)
_______ Focused auditory stimulation: frequent repetition of specific vocabulary
_______ Broad target recasts (using a language recast if the child's speech is relatively clear, and a speech recast if unclear speech is the main issue)
_______ Choices: also known as 'forced alternatives' (e.g. "do you want the APPLE or the BANANA?")

9. Please provide **ONE** key reason for your choice of expressive vocabulary techniques

(this could be based on any of a broad range of factors e.g. the evidence base, your clinical experience, observations, particular aspects regarding how the child responds)

____

10. Are there any techniques for **expressive vocabulary** which you think we've missed? If so, please state (optional)

____

11. Which of the following activities might you choose to incorporate your chosen intervention techniques (for expressive vocabulary) into when delivering this intervention?

(either directly yourself or through the training of a relevant professional/family member)

	Yes	No
Storybooks	○	○
Everyday routines	○	○
Child led play (i.e. no restrictions on what the child plays with or how)	○	○
Turn taking games (e.g. fishing, pop up pirate)	○	○

Electronic (app) games	○	○
Adult directed play (i.e. the adult selects the object or toy and guides how it is played with)	○	○
What's in the bag/box	○	○
Barrier games	○	○
Activities involving posting pictures	○	○
Memory games (e.g. Kim's game)	○	○
Singing/nursery rhymes	○	○
Puzzles	○	○

Using toys to act things out (pretend play)	○	○
Lotto games	○	○
Matching sounds to pictures	○	○
Picture/object sorting	○	○

12. Are there any activities you think we've missed? If so please state below (optional)

____

<u>Survey page 3</u>

<u>Reminder</u>
Age: **3:0-4:11 years**
Profile: **(consistent) phonological SSD** and features of **DLD**.
Intervention: COMBINED to target both **Expressive vocabulary + speech intelligibility.**

Speech intelligibility might be improved through environmental strategies or by developing the accuracy of speech production.

You may think “my answers depend on…”. That’s ok. Just give the answer you think fits best.

lease base your responses on what you would do, rather than on what your service might currently offer.

You are half way through the survey. In the next part, we will be asking you about intervention techniques for speech intelligibility.

13. What environmental strategies might you consider for supporting **speech intelligibility** in the child's everyday environment? (e.g. home, nursery)

	Yes	No
Availability of pictures for the child to refer to (e.g. a picture board at snack time). These might be referred to as communication/aided language/activity boards.	○	○
Encouraging the child to use gesture, sign or 'act out' what they are trying to say	○	○
Asking the child to 'show you' using visual cues in the environment. This might include pointing to an object.	○	○

Personalised communication book/passport containing pictures/symbols	○	○

14. Are there any environmental strategies for **speech intelligibility** which you think we've missed? If so, please state (optional)

____

15. What (input focused) intervention techniques would you consider for improving **speech intelligibility** within this integrated intervention with oral vocabulary?

	Yes	No
Asking the child to identify a word which differs from another by a sound (e.g. maximal or minimal pairs)	○	○
Auditory bombardment (repeatedly presenting target sounds, either in isolation or words containing the sounds)	○	○
Using multi-sensory cues (e.g. drawing attention to the part of your mouth where the sound is formed)	○	○

Speech recasts (repeating back a word to the child with their errors corrected)	○	○
Broad target recasts (using a language recast if the child's speech is relatively clear, and a speech recast if unclear speech is the main issue)	○	○
Asking the child to discriminate between different non speech sounds	○	○

16. Would you prioritise any of **your selected** speech intelligibility techniques over the others?

○ Yes

○ No

Display This Question:

If Would you prioritise any of your selected speech intelligibility techniques over the others? = Yes

Carry Forward Selected Choices from "What (input focused) intervention techniques would you consider for improving speech intelligibility within this integrated intervention with oral vocabulary?"

17. If yes to the above, how would you prioritise your selected techniques?

1-highest priority
_______ Asking the child to identify a word which differs from another by a sound (e.g. maximal or minimal pairs)
_______ Auditory bombardment (repeatedly presenting target sounds, either in isolation or words containing the sounds)
_______ Using multi-sensory cues (e.g. drawing attention to the part of your mouth where the sound is formed)
_______ Speech recasts (repeating back a word to the child with their errors corrected)
_______ Broad target recasts (using a language recast if the child's speech is relatively clear, and a speech recast if unclear speech is the main issue)
_______ Asking the child to discriminate between different non speech sounds

18. Please provide **ONE** key reason for your choice of (input focused) speech intelligibility techniques
(this could be based on any of a broad range of factors e.g. the evidence base, your clinical experience, observations, particular aspects regarding how the child responds)

____

19. Are there any (input focused) techniques for **speech intelligibility** which you think we've missed? If so, please state (optional)

____

20. Would you deem it essential to also incorporate **output focused** techniques into your intervention? (i.e. where the child is expected to **produce** the sound/word)

○ Yes

○ No

21. Please provide **ONE** key reason for your answer to the previous question

____

22. Which of the following activities might you choose to incorporate your chosen intervention techniques (for speech intelligibility) into when delivering this intervention?
(either directly yourself or through the training of a relevant professional/family member)

	Yes	No
Storybooks	○	○
Everyday routines	○	○
Child led play (i.e. no restrictions on what the child plays with or how)	○	○
Turn taking games (e.g. fishing, pop up pirate)	○	○
Electronic (app) games	○	○
Adult directed play (i.e. the adult selects the object or toy and guides how it is played with)	○	○
What's in the bag/box	○	○

Barrier games	○	○
Activities involving posting pictures	○	○
Memory games (e.g. Kim's game)	○	○
Singing/nursery rhymes	○	○
Puzzles	○	○
Using toys to act things out (pretend play)	○	○
Lotto games	○	○
Matching sounds to pictures	○	○
Picture/object sorting	○	○

23. Are there any activities you think we've missed? If so please state below (optional)

____

Survey page 4

Reminder
Age: **3:0-4:11 years**
Profile: **(consistent) phonological SSD** and features of **DLD**.
Intervention: COMBINED to target both **Expressive vocabulary + speech intelligibility.**

Speech intelligibility might be improved through environmental strategies or by developing the accuracy of speech production.

You may think "my answers depend on…". That's ok. Just give the answer you think fits best.

please base your responses on what you would do, rather than on what your service might currently offer.

Nearly there!
This final section of the survey focuses on how you might deliver your intervention techniques.

---

24. Would you aim for any particular aspects of 'dosage' within this intervention?
(e.g. number of sessions or number of times a technique is used in an activity)

○ Yes

○ No

Skip To: Q35 If Would you aim for any particular aspects of 'dosage' within this intervention? (e.g. number of se... = No

---

25. If yes, what aspects of dosage would you have an aim for?

(Reminder: please base your responses on what YOU would do, rather than on what your service might currently offer)

Total number of sessions/teaching episodes (e.g. 8 therapy sessions)	○
Total duration of a single session/teaching episode (e.g. a single session lasts half an hour)	○
Total duration of the intervention (e.g. 2 months)	○
Number of times a TECHNIQUE is used within a single therapy activity (e.g. 50 repetitions of a key sound during a single fishing game)	○

Number of times a TECHNIQUE is used per session/teaching episode (e.g. 100 models of a word over a half hour therapy session)	○
Number of times a TECHNIQUE is used for the duration of the intervention (e.g. 1000 recasts over 6 half hour sessions)	○

26. Please provide **ONE** key reason why you would, or would not, aim for any particular aspects of dosage within this intervention

____

27. Who would you recommend delivers your chosen intervention techniques?

1- most recommended; 5- least recommended

_______ Clinician only
_______ Parent/guardian only (with adequate clinician support)
_______ Education staff only (with adequate clinician support)
_______ Clinician + education staff + parent/guardian (both with adequate clinician support)
_______ Education staff + parent/guardian (both with adequate clinician support)

28. If the technique is delivered through a significant other, what strategies might you use to support them with this?

	Yes	No
Modelling the technique to them	○	○
Discussing a video of someone else doing it	○	○
Video them doing it, then discuss	○	○
Written guidance to take away	○	○
Identifying the best time for them to work with their child within daily life	○	○
Providing picture prompts to put up in home/nursery	○	○

Group training session with other parents	○	○

29. Are there any other strategies you would use to support the 'significant others' technique delivery? Please state here if so (optional)
____

30. Where would you recommend your selected techniques are implemented?
1- most recommended; 7-least recommended

______ Clinic only
______ Home (or when 'out and about' with their family) only
______ Education setting only
______ Clinic + home (or when 'out and about' with their family)
______ Clinic + education setting
______ Clinic + home (or when 'out and about' with their family) + education setting
______ Home (or when 'out and about' with their family) + education setting

31. If you have any other comments, please state below.

Please click the red arrow
____
