# Supplementary material for: Concurrent Targeting of Expressive Vocabulary and Speech Comprehensibility in Pre-Schoolers with Developmental Language Disorder and Phonological Speech Sound Disorder Features: A Survey of UK Practice
Source: Children (Basel). 2025 Nov 18;12(11):1568. doi: 10.3390/children12111568 (PMC12650986; doi:10.3390/children12111568)
Supplement: Supplementary file 1 [file children-12-01568-s001.zip › Supplementary material S4.pdf]

Supplementary material S4:
Example recording from think aloud interviews (Section: target setting)

**Questions**	**Probes**	**Interview 1.**	**Interview 2.**	**Interview 3.**
What would you target to improve **(speech intelligibility/comprehensibility?)** within this combined intervention?	1.Tell me what you are thinking<br><br>2.What does this term mean to you?<br><br>3.Can you put the question into your own words?	Good to remind participants it's about what THEY would do rather than their service.<br><br>Tricky to complete when choosing between 2 important target areas.<br><br>**Action: add in reminder sentence at top about no wrong answers, "we appreciate that it may depend…"**	Good to have recaps on each page<br><br>Didn't understand you have to drag the response (not click). **Action: instruct to do this for the first question involving drag/drop.**<br><br>Complexity of speech/typically developing norms- would depend on the pattern of speech errors. **Action: re-word, their errors may would make the eligible for any of the approaches given.**<br><br>Expressive vocabulary targets fit more easily into a hierarchy.<br><br>Guided by what the child is doing as you go along- has put what you would aim for. **Action: re-word, what would you start**	"How do I do this?" **Action: clarify drag and drop into place.**<br><br>Expressive vocabulary- if apraxia would give a different answer. **Action: reminder that the child has phonological SSD at the start of each section.**

			**with/aim for. It might change as you go along (initially, what would you plan for?).**	

What would you target to improve **expressive vocabulary** within this combined intervention?				
How would you format your speech and vocabulary targets within this combined intervention?				
